# Association of Paroxysmal Versus Persistent Atrial Fibrillation with In-hospital Outcomes and 30-day Readmission After Inpatient Atrial Fibrillation Ablation

**DOI:** 10.19102/icrm.2024.15066

**Published:** 2024-06-15

**Authors:** Min Choon Tan, Yong Hao Yeo, Qi Xuan Ang, Bryan E-Xin Tan, Jian Liang Tan, Pattara Rattanawong, Joaquim Correia, Aneesh Tolat

**Affiliations:** 1 Department of Internal Medicine, New York Medical College at Saint Michael’s Medical Center, Newark, NJ, USA; 2 Department of Cardiovascular Medicine, Mayo Clinic, Phoenix, AZ, USA; 3 Department of Internal Medicine/Pediatrics, Beaumont Health, Royal Oak, MI, USA; 4 Department of Internal Medicine, Sparrow Health System and Michigan State University, East Lansing, MI, USA; 5 Section of Cardiology, Department of Medicine, Baylor College of Medicine, Houston, TX, USA; 6 Department of Cardiovascular Medicine, Hospital of the University of Pennsylvania, Philadelphia, PA, USA; 7 Demoulas Center for Cardiac Arrhythmias, Massachusetts General Hospital, Harvard Medical School, Boston, MA, USA; 8 Department of Cardiovascular Medicine, New York Medical College at Saint Michael’s Medical Center, Newark, NJ, USA; 9 Department of Cardiovascular Medicine, Hartford Healthcare/University of Connecticut, Hartford, CT, USA

**Keywords:** Atrial fibrillation, catheter ablation, cause of readmission, hospital outcomes, paroxysmal atrial fibrillation, persistent atrial fibrillation

## Abstract

Knowledge of the impact of paroxysmal and persistent atrial fibrillation (AF) after catheter ablation on in-hospital outcomes and 30-day readmission remains limited. This study aimed to evaluate the procedural outcomes and 30-day readmission rates among patients with paroxysmal or persistent AF who were hospitalized for AF ablation. Using the Nationwide Readmissions Database, our study included patients aged ≥18 years with AF who were hospitalized and underwent catheter ablation during 2017–2020. Then, we compared the in-hospital procedural outcomes and 30-day readmission rates between patients with paroxysmal and persistent AF, respectively. Our study included 7310 index admissions for paroxysmal AF ablation and 9179 index admissions for persistent AF ablation. According to our analysis, there was no significant difference in procedural complications—namely, cerebrovascular accident, vascular complications, major bleeding requiring blood transfusion, phrenic nerve palsy, pericardial complications, and systemic embolization—between the persistent and paroxysmal AF groups. There was also no significant difference in early mortality between these groups (0.5% vs. 0.7%; *P* = .22). Persistent AF patients had significantly higher rates of prolonged index hospitalization (9.9% vs. 7.2%; *P* < .01) and non-home discharge (4.8% vs. 3.1%; *P* < .01). The 30-day readmission rates were comparable in both groups (10.0% vs. 9.5%; *P* = .34), with recurrent AF and heart failure being two of the most common causes of cardiac-related readmissions. Catheter ablation among hospitalized patients with paroxysmal or persistent AF resulted in no significant difference in procedural complications, early mortality, or 30-day readmission. This suggests that catheter ablation of AF can be performed with a relatively similar safety profile for both paroxysmal and persistent AF.

## Introduction

Atrial fibrillation (AF) is the most common cardiac arrhythmia, with a prevalence of 1%–2% among the United States population.^1,2^ Catheter ablation has proven to be a better rhythm-control strategy than medical therapy alone for the management of AF in several randomized controlled trials.^3–5^ Promising outcomes were observed in both paroxysmal AF (PAF) and persistent AF (PeAF) patients.^6,7^ However, despite the improvement of catheter ablation technologies and procedural safety and efficacy, procedural complications continue to occur with an incidence of 2.9%–5.6%.^8–10^

Clinically, the ablation approach and intraprocedural findings in PAF and PeAF may be different.^11–13^ Patients with PeAF also have more comorbidities compared to patients with PAF.^14^ There are still only limited real-world data on the safety outcomes of ablation of PAF compared to PeAF. Thus, this study aimed to determine and compare the in-hospital procedural outcomes after AF ablation between PAF and PeAF.

## Methods

### Data source

The data were obtained from the National Readmissions Database (NRD) derived from the Healthcare Cost and Utilization Project (HCUP) State Inpatient Databases. The HCUP is sponsored by the Agency for Healthcare Research and Quality. The NRD is one of the United States’ largest publicly available all-payer inpatient care databases. It is an annual database that includes approximately 17 million discharges yearly from 2017–2020. Using verified patient linkage numbers, it can reliably track patient admissions to any hospital in the same state over the course of a year. On the basis of the International Classification of Diseases, Tenth Revision, Clinical Modification (ICD-10-CM) codes, the patient’s diagnoses and procedures during each admission were recorded. We queried this database using ICD-10-CM codes to identify the patient demographic characteristics, the health care facility variables, and the in-hospital outcomes of each admission. Because the NRD is publicly available and de-identified, our study did not require either institutional review board review or informed consent.

### Study population

Using ICD-10-CM codes, we searched for all patients ≥18 years of age with a primary diagnosis of AF (I48.0, I48.1, I48.2) who underwent catheter ablation for AF (02573ZZ, 02563ZZ, 02583ZZ, 025S3ZZ, 025T3ZZ) during their hospitalization from January 2017 to November 2020. We excluded patients who underwent new pacemaker implantation, new implantable cardiac defibrillator implantation, new cardiac resynchronization therapy device implantation, or open surgical ablation, along with any patients with other types of arrhythmias, including supraventricular tachycardia, ventricular tachycardia, ventricular premature complexes, pre-excitation syndromes, and atrial flutter, to ensure a homogenous study population. We also excluded patients with non-specific AF (I48.91) and further divided the included patients into PAF (I48.0) and PeAF (I48.1, I48.2) groups. Patients with missing data for in-hospital mortality and length of stay were also excluded. As the NRD is constructed using a calendar year of discharge data that does not track the patients over the years, index admissions from December were excluded, given that data on 30-day follow-up after discharge would not be available.

### Study endpoints

The primary endpoint of our study was the in-hospital outcomes of patients who were hospitalized and underwent catheter ablation for PAF or PeAF. The outcomes included procedural complications (cerebrovascular accident [CVA], major bleeding requiring transfusion, vascular complications, pericardial complications, systemic embolization, phrenic nerve palsy [PNP]), early mortality (death during index hospitalization and 30-day readmission), length of hospital stay, and discharge disposition. The secondary endpoint of our study was the 30-day readmission rate. The number of days from discharge after index hospitalization to hospital readmission was used to define the time of readmission. If there were multiple readmissions within 30 days after discharge following the index hospitalization, only the first readmission was included for analysis. Same-day transfers within the same hospital or between hospitals were not considered readmissions. The 30-day readmissions following AF ablation procedures were categorized according to the organ system involved and identified by the primary diagnosis for readmission. Identified causes of readmissions included cardiac, respiratory, connective tissue or musculoskeletal, infectious, neurological, gastrointestinal, renal, vascular, endocrinology, and hematology or oncology reasons. Readmissions due to cardiac events were further stratified into AF, congestive heart failure (CHF), ischemic heart disease, arrhythmias other than AF, pericarditis, valvular heart disease, and other non-specified cardiac events.

### Definition of clinical variables

Patient- and hospital-level variables, including age, sex, and hospital characteristics (bed size and teaching status), and patient characteristics (median household income based on zip code, primary payer, type of index admission, and discharge disposition) were derived from NRD variables. Patient comorbidity diagnoses were identified by ICD-10-CM codes. The hospital procedural volume was determined in terms of annual procedural volume quartiles based on cutoffs of the 25^th^, 50^th^, and 75^th^ percentiles.

### Statistical analysis

Continuous data are summarized using mean, standard deviation, median, interquartile range (IQR) (Q1–Q3), and/or range values, with differences between groups tested using the Wilcoxon rank-sum test. Categorical data are summarized as counts and percentages, and differences between groups were tested using Pearson’s chi-squared test. All tests were two-sided, with *P* ≤ .05 indicating statistical significance. Statistical analyses were conducted using Stata version 12.1 (Stata Corporation, College Station, TX, USA). Patients who underwent catheter ablation for PAF and PeAF were stratified based on the occurrence of 30-day readmission. The readmission after discharge following index hospitalization model was run on the patients who survived their index admission. Multivariate predictors of 30-day readmission were determined using binary logistic regression. In multivariate analysis, only the variables with a statistically significant difference in 30-day readmission using the univariate analysis were included. The Cochrane–Armitage test was used to assess the trends of categorical variables, and simple linear regression was used to assess the trends of continuous variables.

## Results

### Study population

Our study included 7310 index admissions (median age, 67 years [IQR, 59–73 years]; 47.1% female) for PAF ablation and 9179 index admissions (median age, 69 years [IQR, 61–75 years]; 37.3% female) for PeAF ablation between January 2017 and November 2020. **Table 1** shows the patient baseline characteristics and hospital characteristics of both groups. Overall, the prevalence rates of reported comorbidities were higher in the PeAF group.

### Procedural outcomes

**Figure 1:**
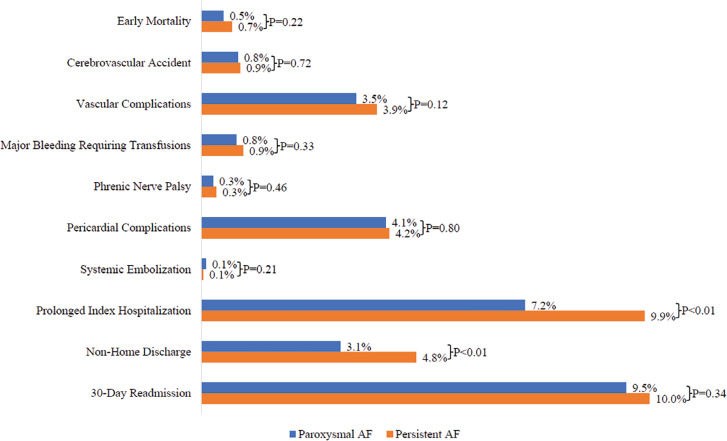
In-hospital outcomes among patients who underwent catheter ablation for paroxysmal or persistent atrial fibrillation.

The procedural outcomes of hospitalized PAF and PeAF patients are depicted in **Figure 1**. Between these two groups (PAF vs. PeAF), there was no significant difference in specific procedural complications—namely, CVA (0.8% vs. 0.9%; *P* = .72), vascular complications (3.5% vs. 3.9%; *P* = .12), major bleeding requiring blood transfusion (0.8% vs. 0.9%; *P* = .33), PNP (0.3% vs. 0.3%; *P* = .46), pericardial complications (4.1% vs. 4.2%; *P* = .80), and systemic embolization (0.1% vs. 0.1%; *P* = .21). There was also no significant difference in early mortality between the two groups (0.7% vs. 0.5%; *P* = .22). Compared to those with PAF, a greater proportion of patients with PeAF had significantly prolonged index hospitalizations (≥7 days) (9.9% vs. 7.2%; *P* < .01) and non-home discharge (4.8% vs. 3.1%; *P* < .01).

### Early readmissions after atrial fibrillation catheter ablation

**Figure 2:**
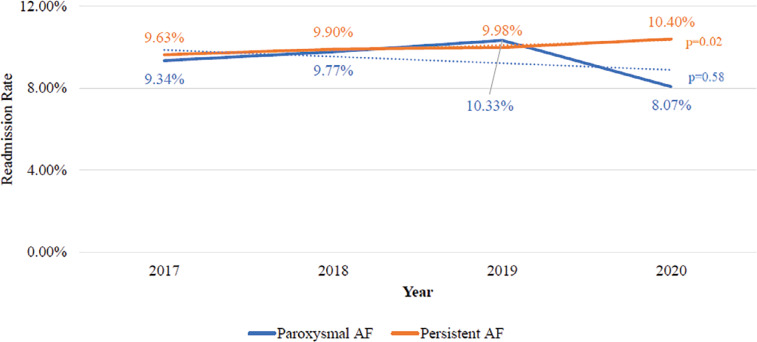
Trend in 30-day readmission rate after catheter ablation for paroxysmal or persistent atrial fibrillation.

**Figure 3:**
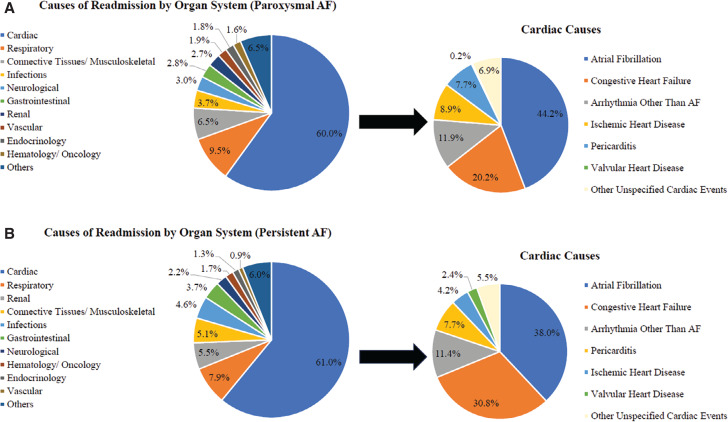
Causes of early readmission (≤30 days) after catheter ablation for **(A)** paroxysmal atrial fibrillation and **(B)** persistent atrial fibrillation.

Among the 16,436 patients who were discharged following catheter ablation, 675 patients with PAF (9.5%) and 894 patients with PeAF (10.0%) were readmitted within 30 days of index hospitalization discharge, and there was no significant difference in their readmission rates (*P* = .34). There was a non-significant decreasing trend in the all-cause 30-day readmission rate from 9.3% in 2017 to 8.1% in 2020 (*P* = .58) among patients with PAF but a significant increasing trend (from 9.6% to 10.4%; *P* = .02) among patients with PeAF **(Figure 2)**. The overall median lengths of stay during readmission in patients with PAF and PeAF were 2 days (IQR, 1–3 days) and 2 days (IQR, 1–4 days), respectively. Cardiac events were the most common cause of 30-day readmission (PAF vs. PeAF, 60.0% vs. 61.8%) in both groups **(Figure 3)**. Additional analysis was performed to determine the etiologies of 30-day readmission among patients who were readmitted for cardiac events. The most common etiologies in cardiac-related readmissions were recurrent AF (PAF vs. PeAF, 44.2% vs. 38.0%) and CHF (PAF vs. PeAF, 20.2% vs. 30.8%). Overall, the early readmission rate due to recurrent AF was 2.3% (PAF vs. PeAF, 2.4% vs. 2.3%; *P* = .41), while that due to CHF was 1.5% (PAF vs. PeAF, 1.1% vs. 1.8%; *P* < .01).

### Predictors of 30-day readmission following catheter ablation for paroxysmal and persistent atrial fibrillation

Through multivariate analysis, female sex (PAF adjusted odds ratio [OR], 1.28; *P* = .01 and PeAF OR, 1.27; *P* < .01), chronic kidney disease (CKD) (OR, 1.32; *P* = .02 and OR, 1.42; *P* < .01), chronic obstructive pulmonary disease (COPD) (OR, 1.66; *P* < .01 and OR, 1.31; *P* < .01), diabetes mellitus (DM) (OR, 1.32; *P* < .01 and OR, 1.39; *P* < .01), and a prolonged index hospital stay of >7 days (OR, 1.62; *P* < .01 and OR, 1.45; *P* < .01) were identified as the independent predictors of 30-day all-cause readmissions in both patient groups **(Table 2)**. For patients with PAF, chronic liver disease (OR, 1.67; *P* = .04) was the additional independent predictor of readmissions. Age (OR, 1.01; *P* = .02) and anemia (OR, 1.71; *P* < .01) were the additional independent predictors of readmissions, while centers with a high procedural volume (fourth quartile) are associated with a lower odds of early admissions (OR, 0.71; *P* < .01) for patients with PeAF.

### Timing of readmission from index discharge

The median timing of readmission among all-cause early readmissions was 10 days (IQR, 4–18 days) from index discharge. Overall, 43.1% of readmissions occurred within 1 week of discharge, while 67.4% occurred within 14 days of discharge.

## Discussion

To our knowledge, this study is the largest all-payer data analysis reporting the impact of PAF versus PeAF on short-term outcomes following catheter ablation for AF among hospitalized patients in the United States. Our data analysis showed that: (1) there is no significant difference in procedural complications or 30-day readmission rates after catheter ablation between PAF and PeAF among hospitalized patients; (2) the PeAF group has a higher rate of prolonged index hospitalization and non-home discharge; (3) approximately 10% of patients with PAF and PeAF were readmitted within 30 days of discharge from the index admission after AF ablation; (4) cardiac-related events were the most common reasons for 30-day readmission after AF ablation for both groups (more than half of them were due to recurrent AF and CHF); (5) female sex, CKD, COPD, DM, and prolonged index hospitalization appeared to be independent predictors of 30-day readmission after AF ablation in both groups; and (6) chronic liver disease among PAF patients and age and anemia among PeAF patients, respectively, were additional predictive factors of 30-day readmission.

Our study showed that the rates of procedural complications were similar among patients with PAF and PeAF who underwent AF ablation. This finding is consistent with that of the previously published studies by Willy et al. and Yang et al.^15,16^ Other than pulmonary vein isolation (PVI) alone, additive strategies, including linear radiofrequency lesions, posterior wall isolation, and complex fractionated electrograms, are often used in patients with PeAF.^17,18^ The adoption of these approaches pre-emptively in patients with PeAF remains debatable due to their potential safety and beneficial concerns. A study that was conducted to assess the safety profile of these strategies reported no significant differences in the procedural outcomes compared to PVI alone.^13^ Similarly, our findings revealed no statistical significance in the procedural complications between the two groups, although the PeAF group may often receive additional additive ablations other than PVI alone. Nonetheless, while the early mortality rates reported were similar in both groups, the rate reported in our analysis is higher than that reported in existing studies, which could be attributed to a sicker patient population who required admission for AF and underwent an inpatient ablation procedure.

Nonetheless, patients with PeAF have greater risks of more prolonged hospitalization (≥7 days) and non-home discharge after AF ablation. Similar to the previously published studies, our study showed that the reason for this could be that patients with PeAF are often older and have more comorbidities compared to patients with PAF.^19^ Other reasons could be that differences in ablation strategy in patients with PeAF (such as the addition of posterior wall isolation) may result in a longer procedural time and greater fluid administered during ablation, which can lead to volume overload and, therefore, a greater time required for diuresis post-ablation.^13^

In our study, 10% of the patients in both groups were readmitted within 30 days after AF ablation, while 2.3% were readmitted for recurrent AF. While the all-cause readmission rate in our study was comparable to that in previously published studies, the 30-day readmission rate due to recurrent AF in this study was lower than the 3.3% rate reported by Freeman et al. between 2010 and 2014.^20^ The difference in the readmission rate for recurrent AF might be due to improvements in ablation strategies, tools, or techniques in the contemporary era. While our data suggested that 2.3% of the patients had early AF recurrence during the blanking period, which required inpatient management, the actual AF recurrence rate during the blanking period might have been underestimated in our study as the NRD does not include patients with recurrent AF who were managed as outpatients. Studies showed that AF recurrence could occur at rates of 8.6% and 22.3% during 3-month and 1-year follow-up, respectively.^21,22^ Most early recurrences of AF occur within the first 2 weeks after AF ablation,^23–25^ and possible mechanisms include temporary left atrial inflammation and conduction recovery at the ablation sites.^23–26^ While PeAF has a higher rate of AF recurrence compared to PAF after ablation in long-term follow-up, there is no significant difference in 30-day readmissions due to recurrent AF between the two groups in our cohort.

The AF ablation procedure can restore and maintain sinus rhythm. This could improve the atrial systolic function and increase left ventricular filling.^27,28^ Multiple trials have shown promising results in the endpoints with catheter ablation among patients with CHF.^4,29,30^ While this is ideal, a study by Huang et al. showed that 12.6% of the patients who underwent AF ablation developed symptomatic CHF requiring prolonged index hospitalization or readmission.^31^ CHF was the second-most common cause of cardiac-related readmissions in our study. This may be due to volume overload following an AF ablation procedure or could also be related to the recurrence of AF with a rapid ventricular rate, resulting in decompensated heart failure. CHF readmissions were greater in patients with PeAF, likely due to a higher prevalence of CHF comorbidities and a greater risk of arrhythmia-induced cardiomyopathy in those patients.^32,33^ The higher CHF-related readmission rate observed could also be attributed to a larger ablative lesion set and longer procedural time during the ablation procedure for PeAF.

Early readmission has been suggested as a risk factor for mortality within 2 years of discharge.^34^ Our study revealed several factors that predict 30-day readmission after AF ablation in both PAF and PeAF patients. This provides a reference in patient selection in the shared decision-making process and emphasizes closer monitoring postprocedurally in those high-risk patients.

### Limitations

Despite routine quality-control measures by HCUP to ensure data validity and reliability, there are still some limitations in our study. First, the procedural outcomes analyzed are limited to those in hospitalized patients. The lack of inclusion of AF procedures performed at outpatient settings might lead to some extent of study bias. Thus, our findings might not apply to the population that underwent AF ablation at outpatient settings. Second, as with most of the large administrative database studies, the main limitation includes miscoding in primary diagnoses and under-reporting of secondary diagnoses. Any out-of-hospital deaths that occurred prior to readmission were not recorded, which limits our early mortality to in-hospital mortality. Third, specific patient variables—such as left ventricular ejection fraction; medications (like anti-arrhythmic agents or diuretics); and procedural characteristics like the type of anesthesia, procedural duration, ablative strategy, and lesion set and location—were not available. This limits our attempts to explore the impact of AF catheter ablation on the procedural outcomes. Fourth, the exclusion of patients with a non-specific AF diagnosis in our study could result in under-representation of the PAF cohort. The final limitation of our study is not being able to track the patients who were admitted in one state and readmitted in another state.

## Conclusion

Catheter ablation of AF in both hospitalized PAF and PeAF patient groups led to no significant difference in procedural complications, early mortality, or 30-day readmission, but higher rates of prolonged hospital stay and non-home discharge in the PeAF group were observed. The 30-day readmission rate was comparable in both groups, and cardiac reasons (recurrent AF and CHF) were the main causes of early readmission post-AF ablation. Our data suggest that patients with AF who undergo catheter-based ablation may have a similar safety profile and in-hospital clinical outcomes regardless of the AF type.

## Figures and Tables

**Table 1:** Baseline Patient and Hospital Characteristics for Patients Who Underwent Catheter Ablation for Paroxysmal or Persistent Atrial Fibrillation

	Catheter Ablation for Paroxysmal AF		Catheter Ablation for Persistent AF		*P* Value
	n	%	n	%	
No. of admissions	7310	44.3%	9179	55.7%	
Baseline characteristics					
Age, mean (SD), years	65.69 (11.25)		67.89 (10.54)		<.01
Female sex	3444	47.1%	3419	37.3%	<.01
Anemia	155	2.1%	217	2.4%	.30
CKD	832	11.4%	1538	16.8%	<.01
Chronic liver disease	126	1.7%	219	2.4%	<.01
COPD	1325	18.1%	1905	20.8%	<.01
Coagulation disorder	250	3.4%	389	4.2%	<.01
CHF	1938	26.5%	4560	49.7%	<.01
Coronary artery disease	2041	27.9%	2961	32.3%	<.01
DM	1688	23.1%	2360	25.7%	<.01
Hyperlipidemia	3890	53.2%	5099	55.6%	<.01
Hypertension	5230	71.6%	7287	79.4%	<.01
Obesity	1721	23.5%	2738	29.8%	<.01
Obstructive sleep apnea	1841	25.2%	2758	30.1%	<.01
Peripheral arterial disease	771	10.6%	1273	13.9%	<.01
Prior coronary artery bypass graft	366	5.0%	521	5.7%	.06
Prior implantable cardioverter-defibrillator placement	338	4.6%	700	7.6%	<.01
Prior myocardial infarction	526	7.2%	707	7.7%	.22
Prior pacemaker placement	717	9.8%	1056	11.5%	<.01
Prior percutaneous coronary intervention	744	10.2%	962	10.5%	.53
Prior stroke/transient ischemic attack	624	8.5%	793	8.6%	.82
Pulmonary hypertension	291	4.0%	633	6.9%	<.01
Valvular heart disease	1078	14.8%	1942	21.2%	<.01
CHA_2_DS_2_-VASc score, mean (SD), points	2.55 (1.50)		2.87 (1.51)		<.01
Hospital variables					
Hospital size					<.01
Small	350	4.8%	503	5.5%	
Medium	1987	27.2%	2727	29.7%	
Large	4973	68.0%	5949	64.8%	
Hospital procedural volume					<.01
First quartile	1613	22.1%	2262	24.6%	
Second quartile	1558	21.3%	2369	25.8%	
Third quartile	1869	25.6%	2346	25.6%	
Fourth quartile	2270	31.1%	2202	24.0%	
Prolonged index hospital stay (length of stay, ≥7 days)	527	7.2%	906	9.9%	<.01

*Abbreviations:* AF, atrial fibrillation; CHF, congestive heart failure; CKD, chronic kidney disease; COPD, chronic pulmonary disease; DM, diabetes mellitus; SD, standard deviation.

**Table 2:** Multivariate Independent Predictors of 30-day Readmissions After Catheter Ablation for Paroxysmal or Persistent Atrial Fibrillation

Predictors of 30-day Readmission	30-Day Readmissions After Catheter Ablation for Paroxysmal AF				30-Day Readmissions After Catheter Ablation for Persistent AF			
	Adjusted OR	Lower Limit	Upper Limit	*P* Value	Adjusted OR	Lower Limit	Upper Limit	*P* Value
Female sex	1.28	1.07	1.53	.01	1.27	1.09	1.49	<.01
CKD	1.32	1.05	1.67	.02	1.42	1.19	1.69	<.01
COPD	1.66	1.37	2.01	<.01	1.31	1.11	1.54	<.01
DM	1.32	1.09	1.60	<.01	1.39	1.19	1.63	<.01
Prolonged index hospital stay (length of stay, ≥7 days)	1.62	1.23	2.12	<.01	1.45	1.17	1.79	<.01
Chronic liver disease	1.67	1.02	2.74	.04	–	–	–	–
Hospital procedural volume								
First quartile	–	–	–	–	–	–	–	–
Second quartile	–	–	–	–	0.99	0.82	1.20	.95
Third quartile	–	–	–	–	1.02	0.84	1.24	.82
Fourth quartile	–	–	–	–	0.71	0.56	0.90	<.01
Age	–	–	–	–	1.01	1.01	1.02	.02
Anemia	–	–	–	–	1.67	1.18	1.69	<.01

*Abbreviations:* AF, atrial fibrillation; CKD, chronic kidney disease; COPD, chronic pulmonary disease; DM, diabetes mellitus; OR, odds ratio.
